# Mosquito-Borne Diseases: Social Representations of a University Community in Endemic Outbreaks

**DOI:** 10.3390/idr13020047

**Published:** 2021-05-30

**Authors:** Sylvain Delouvée, Gail Moloney, Kathleen McColl, Grégory Lo Monaco

**Affiliations:** 1 Department of Psychology, Université Rennes 2, LP3C–EA 1285, 35000 Rennes, France; 2 School of Health and Human Sciences, Southern Cross University, Military Rd, East Lismore, NSW 1235, Australia; gail.moloney@scu.edu.au; 3 School of Public Health–École des Hautes Études en Santé Publique, 35000 Rennes, France; kathleen.mccoll@eleve.ehesp.fr; 4 Department of Social Psychology, Aix Marseille Université, ADEF–EA 4671, 13013 Marseille, France; gregory.lo-monaco@univ-amu.fr

**Keywords:** social representations, emerging infectious diseases, mosquito-borne diseases, health-protective behaviors

## Abstract

(1) Background: Studying social representations as lay theories allows for a better understanding of the common sense knowledge constructed around mosquito-borne diseases and the impact this may have on attitudes and behaviors. (2) Methods: A hierarchical evocation questionnaire was circulated through an Australian academic community and analyzed by prototypical analysis and correspondence factor analysis. (3) Results: Representational areas are regulated by participant age and whether or not they had contracted a mosquito-borne disease. (4) Conclusions: Collecting and understanding social representations has the potential to help social actors implement strategies that encourage people to access information and adopt behaviors in line with the scientific reality of the phenomenon, rather than limiting lay theories.

## 1. Introduction

With the current pandemic, emerging infectious diseases (EIDs) form a central part of everyone’s news and daily life. An EID is a label attached to various new or re-emerging diseases (COVID-19, Zika, Chikungunya, HIV/AIDS, avian flu). Although we are not specialists in virology or epidemiology, we note that most of these infectious diseases are known by the general public, primarily through the dissemination of information and images through the press, radio and television. Linked to collective fears [1], these EIDs mobilize an imagination, linking them to beliefs and social representations associated with contagion, therapeutic treatment, affected populations, and science, etc. The media has also seized on these new diseases, with digital social networks disseminating possible public health prevention messages [2] as well as fake news [3,4].

Many studies have focused on the link between knowledge, attitudes, and practices in relation to mosquito-borne diseases: Chikungunya virus [5,6], dengue [7], malaria (especially in India due to the widespread nature of this disease [8,9,10,11]) or Zika virus [12,13]. The aim of these studies is to show the link between prevention practices, knowledge of specific diseases (infection, transmission, protection, care, etc.) and the attitudes towards them. Two of these studies have focused on regions within Australia. A study of knowledge and attitudes towards mosquitoes and mosquito-borne diseases was conducted on a representative sample of the Western Australian population (Perth, Peel, Southwest, etc. [14]). The results of this study indicated that the majority of people surveyed have better knowledge about the Ross River virus than other endemic diseases. This knowledge was less developed in the younger age group. A second study involved approximately 500 persons living in Queensland [15]. Results show that concern about disease was a significant predictor of mosquito breeding site removal and that raising concerns about these diseases can increase the use of personal prevention strategies.

Social representations theory (SRT) [16] allows us to understand how a new, unfamiliar, or threatening issue or social object is grasped by groups in order to make it meaningful. Social representations draw on lay thinking (i.e., common sense theory) which is differentiated not only from expert thinking but from the mass media response. Social representations can be defined as “systems of opinions, knowledge, and beliefs particular to a culture, a social category, or a group associated with objects in the social environment” (p. 478, [17]). Social representations, in the same way as any other form of social thinking [18,19], suggest that a cognitive activity or its apparent result is more related to a specific social and cultural affiliation than to the supposedly objective properties of the information to be processed [17]. SRT is particularly concerned with the transformation that occurs as knowledge moves from the scientific universe into common sense [20]. In the area of health, for example, individual and collective behaviors make sense of what may be considered irrational or absurd in terms of medical epidemiological logic.

Among the different approaches developed in line with SRT, the structural model focuses on the internal structure of social representations, establishing a differentiation between the core (stable) and peripheral (fluid) aspects of the representation. More precisely, according to Abric [21], elements in a representation do not have the same functions nor do they possess the same characteristics. The central elements define the identity of the social representation (from the point of view of meaning and organization) and the peripheral elements allow for the adaptation of the representation to various social contexts.

Early research investigating the social representations of health and illness [22] highlights how the beliefs associated with emerging social issues serve as functions for the group that sustains the existence of those beliefs [23]. Indeed, social representation’s sense-making and identity functions can highlight the social psychological issues associated with EID and mosquito-borne diseases (MBDs) in particular. EIDs are novel and threatening events, and studying the emergence and spread of social representations [23] associated with MBDs can help to increase our understanding of the public’s apprehension towards the potential dangers associated with EIDs. As Joffe [24] points out: “The fundamental contribution of social representation theory to the health psychology field is its ability to enhance understanding of how lay people make meaning of facets of health and illness, and of how these meanings evolve.” (p. 560) Studying social representations as lay theories will allow for a better understanding of the common sense constructed around mosquito-borne disease and the impact this may have on attitudes and behaviors.

## 2. Materials and Methods

Data were collected by means of an online questionnaire disseminated to staff and students through the intranet at a University in NSW on the east coast of Australia. One hundred and thirty staff and students from the Southern Cross University (New South Wales region, Australia) participated in the study. This sample was sufficient with regard to the literature for carrying out the analyses presented below [25] and also in comparison with similar studies of other EIDs [26,27,28]. This Australian university community was chosen because it is located in a region of Australia that has not been considered by previous MBD studies [14,15]. In addition, a viral outbreak of Ross River had occurred a few weeks earlier in this region. The Ross River virus is an arbovirus (arthropod-borne virus) endemic in Australia and Papua New Guinea that causes fever, rash and myalgia in affected people [29]. Ross River virus infections occur sporadically in Australia. This local outbreak reignited the risk of MBDs to the public, promoted through public health prevention messages. The object of representation was, again, a source of media and inter-individual discussions. Social representations are characterized by a function of orienting behaviors and practices, therefore, the emergence of this local outbreak of Ross River in this part of Australia provided an ideal context for a study of lay thinking about MBDs.

The mean age of participants was 40.2 years old (*SD* = 14.4, range = 17–69). There were 28 men, 101 women and one participant who did not give their gender. Only 6.92% have ever contracted an MBD before, but 46.92% had a close family member or a close friend who had contracted an MBD. It should be noted that the population was relatively homogeneous (e.g., similar socio-economic or socio-cultural levels). The survey was anonymous and confidential.

The questionnaire was presented as part of an international study seeking to understand certain opinions about how we view the world. Participants were asked to fill in a hierarchical evocation questionnaire [30,31]. This method is based on free association and consists of a stimulus word (here “mosquito-borne disease”) to which participants are asked to associate spontaneously four words, phrases or feelings. Participants were then asked to rank the four words or phrases from the most important (1) to the least (4). Finally, participants were asked demographics questions (age, gender) and whether they had been exposed to an MBD.

## 3. Results

The corpus is composed of 130 participant responses. The responses were categorized by the authors, independently, and using classical rules of content analysis [32,33,34]. This categorization resulted in 163 different categories (109 are hapax, association produced by a single participant, 66.87% of this corpus).

On the basis of the associated and ranked words, a prototypical analysis [25] was performed using a cross-table which included the frequency and average importance criteria of induced words (cf. Table 1). This allowed us to highlight the salience of the elements in the representation by crossing two independent criteria: the frequency of appearance of an element and its rank of appearance.

Firstly, two sub-categories were revealed by crossing both high frequency and importance. The first sub-category appears to refer to the different MBD types (e.g., “malaria”, “Ross River”, “dengue”), whereas the second one described the characteristics of these diseases (e.g., “blood”, “illness”). These elements, which potentially constitute the central core of the social representation of MBD [25,32,35,36], refer to the descriptive aspects of representational content.

Malaria was to be the most prototypical and well-known MBD for this sample of Australians, despite the fact it is not the most prevalent disease amongst this population [37]. Ross River virus was less associated but considered as one of the most important by the participants reflecting the participants’ familiarity with this disease. Experience appears to be linked to the risk of contracting this disease, relative to the other elicited words such as “water”.

The other cells of the table are also interesting from a descriptive point of view. In the top right cell, we find associations that are less frequent, but which appeared in the first elicitations. These elements correspond to what Abric [30] calls the "first periphery". These are the most salient elements of the peripheral system of the representation. The bottom left cell groups together the "contrasting" elements [30] that do not have a consensus but are very accessible. Finally, the last cell of the table groups the least-salient associations (the second periphery).

In order to explore the correspondences between the word associations and the characteristics of our sample, all the collected content was submitted to a correspondence factor analysis (CFA) [38]. This analysis highlights differences in terms of frequencies, according to participants’ demographics. The outcome in the form of a graphical representation summarizes the data and reveals how the socio-representational universes are linked to the variables studied. It also enables the identification of the most significant factorial axes [39], and emphasizes the correspondences between the modalities of the independent variables and the words or phrases participants associated with them.

This analysis was performed on associations where the frequency of elicitation was greater than 4 (N = 30, 87.10% of the corpus without hapax). Results showed that having contracted the virus, or knowing someone (family or friend) who had contracted an MBD, and experience (operationalized as age with a median of 40.5), allowed the sample to be divided into two groups of 65 participants. Factor 1 represented 57.59% of inertia and factor 2 represented 42.41%.

**Figure 1:**
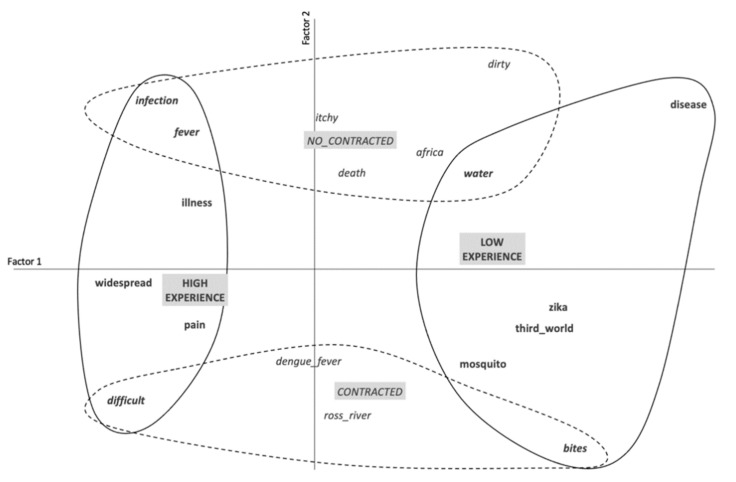
Graphical representation of the results obtained by means of the CFA concerning factors 1 (57.59% of inertia) and 2 (42.41% of inertia).

Drawing from the work of Piermattéo et al. [40], and in line with the theoretical tenets of Deschamps [41], the variables which contribute to the formation of the first factor were “High Experience” (i.e., older people) and the “Low Experience” (i.e., younger people) variables: CF (High Experience) = 0.48 + CF (Low Experience) = 0.50, thus a total contribution of 98% for the formation of factor 1. Factor 2 was formed by the contribution of the “Not contracted” and the “Contracted” variables: CF (Not contracted) = 0.50 + CF (Contracted) = 0.47, thus a total contribution of 97% for the formation of factor 2. Figure 1 of the correspondence factor analysis represents the socio-representational universes specific to each factor. Factor 1 (horizontal axis) is structured by contrasting people according to their experiences. People with a high level of experience (the oldest) evoke more frequently than others the elicitations: “fever”, “pain”, “infection”, which were directly linked to consequences of the disease. This result may be explained by the collective memory of previous mosquito-borne epidemics in Australia, or perhaps simply by the fact that, being older, this age group was more likely to have been exposed to the risk of MBDs. In contrast, the socio-representational universe of the people with a low level of experience (the youngest) portrays mosquito-borne diseases through descriptive elements such as “mosquito”, “bites” and “disease”. It is interesting to note that the frequency of the response “Zika” may be explained by the extensive media coverage of that particular epidemic at the time.

*Note 1:* Grayed blocks refer to the variables. “**Variables**” contribute to the formation of factor 1; *“Variables”* refer to the variables which contribute to the formation of factor 2; “***Variables***” refer to the variables which contribute to the formation of both factors 1 and 2. “**Observations**” refer to the observations which contribute to the formation of factor 1; “*Observations*” refer to the observations which contribute to the formation of factor 2; “***Observations***” refer to the observations which contribute to the formation of both factors 1 and 2.

*Note 2:* Factor contributions (FC) are as follows: first factor "High Experience" (FC = 0.48) and "Low Experience" (FC = 0.50); second factor "Uncontracted" (FC = 0.50) and "Contracted" (FC = 0.47).

*Note 3:* The eigenvalues indicate how much of the total Phi2 of our table is accounted for by each factor. The Phi2 is a coefficient derived from the Chi2 which is equal to the Chi2 on the total number of people in the table, and which refers to what is called the inertia. The interpretation of the total Phi2 poses problems [41], so we calculated the percentage of inertia explained by each extracted factor.

Moreover, for “High Experience” participants, associations seem to relate to the risks and dangers of contracting an MBD. This can be explained by the fact that they may feel more vulnerable concerning the consequences associated with this issue. For “Low Experience” people, the analysis revealed associations centered on something which is more distant with a feeling of “not being directly concerned”. This suggested the perceived threat which is more salient for “High Experience” participants than for “Low Experience” ones.

The second factor separates those who had directly contracted an MBD, or who knew a close friend or family member who had contracted this type of disease, from those who had not been in direct contact with someone who had been infected by an MBD. The former group spontaneously mentioned the name of widespread vector-borne disease (such as dengue fever), as well as an endemic Australian disease, Ross River fever. In addition, contracting MBDs appears to highlight participants’ experience and practices related to local diseases and concern about the threat. On the other hand, not having contracted an MBD led to participants’ representation of these diseases as something which is foreign. Joffe [42] describes how risks are often projected onto exogroups, notably as a justification for their inferiority [43]. Eicher et al [44] showed that individuals who think the world is a dangerous place perceive the origin of an EID to be more the result of a large conspiracy or outside groups, rather than a natural cause.

## 4. Discussion

This research provided important insights into how an Australian academic community integrates MBD into their everyday thinking. The first results therefore revealed representational areas, which were regulated by participant age/experience, and whether or not they had contracted an MBD. The anchoring process described by Moscovici [16] accounts for the way in which an object will find its place in the pre-existing thought system of individuals and groups. Thus, we understand the important weight of factor 1: different age groups and, therefore, groups with different experiences, perceive the object in a different way because of their more established frames of thought. The first analysis shed light upon areas of knowledge that were more or less shared by the different groups. In order to study the role of social representations in guiding behavior, and to evaluate participant engagement in adopting measures promoted during prevention campaigns to reduce the spread of MBDs, socio-representational content should be systematically collected prior to studying health-protective behaviors. Thus, collecting and understanding social representations will help social actors implement strategies that encourage people to access information in line with the scientific reality of the phenomenon, rather than from limiting lay theories. Social representations theory complements the KAP (knowledge, attitude, practices) studies that are very common in the health field. Experimental studies have shown how social representations can influence attitudes but also, importantly, that attitudes do not influence social representations [45]. Similarly, Rateau [46] has shown experimentally the hierarchy between social representations and attitudes, adding to the body of early research that investigated the impact of social representations on behaviors [47,48].

The fact that young people’s social-representational universe regarding MBDs is still very descriptive and at a more distant level, reflecting a feeling of “not being directly concerned”, confirms that young people are a risk group for MBDs. Future health communication should specifically target this age group in order to raise awareness and knowledge of the risks associated with mosquitoes. In the absence of vaccines or treatments for MBDs, prevention is essential in order to address common-sensical understanding which, despite their importance in terms of adaptation, may led people to engage in risky behaviors.

## Figures and Tables

**Table 1:** General results in terms of frequency and average importance associated with the categories of words reported by participants.

		Average Importance					
		≤2.5			>2.5		
			*n*	*M*		*n*	*M*
Frequency	≥10%	Malaria Ross River Dengue Blood Illness Water	62 35 24 12 11 10	1.5 2.4 2.5 2.3 1.9 2.3	Death Tropics Swamp Mosquito Sick Africa Itchy Fever Third World	21 16 16 14 14 11 10 10 10	2.9 2.8 2.9 2.9 2.6 3.2 2.9 2.9 2.6
	<10%	Pain Asia Disease Prevent Dirty Infectious Overseas	7 6 5 4 4 4 4	2.3 2 2.4 1.5 2 1.8 2.5	Fear Poverty Widespread Zika Dangerous Bites Infection	7 7 6 5 5 5 4	2.7 2.7 2.8 2.8 3.4 3 3

## Data Availability

Data and materials are available on OSF.
